# 

**Published:** 1902-04-15

**Authors:** 


### CONTENTS.

Mistakes and Failures in Crown and Bridge-work,
*By F. E. Logan, D.D.S.* 163

Dentistry as a Specialty of Medicine,

*By Dr. O. N. Heise, M.D., D.D.S.* 183

Prosthetic Dentistry, - - *By A. G. Rose, D.D.S.* 196

The Effects of Dental Vulcanite upon Certain Metals - 203

The Obtunding of Dentin - - - - - - - - 206

Editorial, - - - - - - - - - - 208

Announcements, - - - - - - - - 209

Indents, - - - - - - - - - - 211

